# Toxic Metabolites and Inborn Errors of Amino Acid Metabolism: What One Informs about the Other

**DOI:** 10.3390/metabo12060527

**Published:** 2022-06-08

**Authors:** Namgyu Lee, Dohoon Kim

**Affiliations:** Department of Molecular, Cell and Cancer Biology, University of Massachusetts Medical School, Worcester, MA 01605, USA; namgyu.lee@umassmed.edu

**Keywords:** inborn error of metabolism (IEM), metabolism, metabolites, toxic metabolites, ammonia, hyperammonemia, methylmalonic acidemia, intoxification

## Abstract

In inborn errors of metabolism, such as amino acid breakdown disorders, loss of function mutations in metabolic enzymes within the catabolism pathway lead to an accumulation of the catabolic intermediate that is the substrate of the mutated enzyme. In patients of such disorders, dietarily restricting the amino acid(s) to prevent the formation of these catabolic intermediates has a therapeutic or even entirely preventative effect. This demonstrates that the pathology is due to a toxic accumulation of enzyme substrates rather than the loss of downstream products. Here, we provide an overview of amino acid metabolic disorders from the perspective of the ‘toxic metabolites’ themselves, including their mechanism of toxicity and whether they are involved in the pathology of other disease contexts as well. In the research literature, there is often evidence that such metabolites play a contributing role in multiple other nonhereditary (and more common) disease conditions, and these studies can provide important mechanistic insights into understanding the metabolite-induced pathology of the inborn disorder. Furthermore, therapeutic strategies developed for the inborn disorder may be applicable to these nonhereditary disease conditions, as they involve the same toxic metabolite. We provide an in-depth illustration of this cross-informing concept in two metabolic disorders, methylmalonic acidemia and hyperammonemia, where the pathological metabolites methylmalonic acid and ammonia are implicated in other disease contexts, such as aging, neurodegeneration, and cancer, and thus there are opportunities to apply mechanistic or therapeutic insights from one disease context towards the other. Additionally, we expand our scope to other metabolic disorders, such as homocystinuria and nonketotic hyperglycinemia, to propose how these concepts can be applied broadly across different inborn errors of metabolism and various nonhereditary disease conditions.

## 1. Introduction

There are roughly 1653 metabolic enzymes encoded in our genome [1], involved in the breakdown, production, or interconversion of various small molecules referred to as ‘metabolites’. Metabolites include building blocks such as amino acids and nucleotides, signaling agents such as sphingolipids or hormones, and the numerous intermediates formed during their formation, breakdown, or interconversion.

When a loss of function mutation in a metabolic enzyme occurs, this can result in an inborn error of metabolism (IEM). There are numerous different IEMs, in diverse metabolic pathways, and the collective incidence of all IEMs is thought to be higher than 1/1000 [2]. The pathologies manifested for each IEM can vary widely and can even vary significantly patient by patient for the same IEM [3,4]. The majority of these disorders are inherited in an autosomal recessive manner, consistent with most mutations causing a loss of enzyme activity, and the disease only manifests when both copies are impaired [2].

In the IEMs, the impairment in enzyme activity should have two potentially harmful consequences: (1) the enzyme cannot make its product, and (2) the substrate of the enzyme will accumulate. If the pathology of an IEM is due to a lack of product, it can be assumed that supplementing the product itself or somehow compensating for the loss of product may have therapeutic benefits. An example of an IEM pathology being caused by loss of enzyme product would be glutathione synthetase deficiency, in which the enzyme glutathione synthetase (GSS), required to produce the antioxidant glutathione, is impaired [5,6,7]. The main symptom of this disorder is hemolytic anemia [8]. It is postulated that pathology occurs due to cells lacking antioxidant capacity, and supplementation with antioxidants vitamin C and E has been suggested [9]. A second example is creatine deficiency syndrome, caused by defects in guanidinoacetate methyltransferase (GAMT), whose product is creatine [10,11]. The disorder manifests as various neurological symptoms, including mental retardation and seizures. Importantly, patients showed improvement upon supplementation of creatine, demonstrating that the pathology is at least in part due to a depletion of creatine [10].

**Figure 1:**
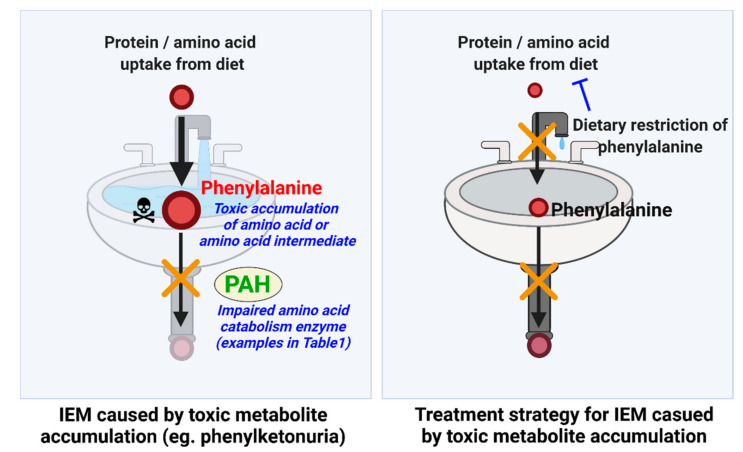
Kitchen sink model for IEM caused by toxic metabolite accumulation. As the majority of IEMs appear to be caused by the accumulation of the substrate of the enzyme that is impaired by the underlying mutation, the pathology and treatment can be conceptualized as a sink. Using phenylketonuria as an example, the loss of function mutation in phenylalanine hydroxylase leaves individuals unable to metabolite phenylalanine; thus, the drain of the sink is blocked and leads to an overflow of phenylalanine in the individual and accumulation of toxic phenylalanine byproducts. Importantly, dietary restriction of phenylalanine prevents the pathology, just as turning off the faucet prevents an overflow. This general sink mechanic appears to be in play in the majority of amino acid breakdown disorders, as described in this review. PAH: phenylalanine hydroxylase.

On the other hand, many IEMs appear to be caused by the excessive accumulation of enzyme substrates [3,4]. As an example, phenylketonuria (PKU) is the most prevalent IEM and is an autosomal recessive disorder caused by the loss of phenylalanine hydroxylase (PAH) activity, the first step in phenylalanine catabolism. In this disorder, a massive accumulation of phenylalanine and phenylalanine byproducts is detected in the plasma, and the disease has varying degrees of severity that is dependent on early intervention. Because the mother’s body is able to catabolize phenylalanine, infants with PKU are normal at birth, but treatment must be initiated upon birth. In severe cases without intervention, significant impairments to brain development occur, and severe neurological symptoms and cognitive impairments can occur [12,13,14]. To a degree, tyrosine is conditionally essential in PKU patients because it cannot be synthesized as a downstream product of phenylalanine breakdown. Importantly, restricting phenylalanine from the diet through a strict regimen results in a remarkable degree of symptom prevention [15,16,17]. This further highlights the importance of early diagnosis, and indeed the national newborn screening programs cover PKU and other disorders in all newborns born in the US [18]. This provides clear evidence that the toxicity is due to substrate accumulation rather than a deficiency of PAH enzyme products. Furthermore, this disease indicates that either phenylalanine or phenylalanine byproducts that form under high concentrations are de facto toxins at the levels that accumulate in the disorder. A useful visual analogy for PKU and for many inborn errors of amino acid metabolism is a kitchen sink (Figure 1): the PAH enzyme, or the various other enzymes that are mutated in the various IEMs, act as drains. Their loss—the clogged drain—causes a buildup of their substrates to toxic levels, overflowing the sink. While ideally, one would want to fix the enzyme defect, i.e., unclog the drain, an effective alternative therapeutic solution is to turn down or turn off the faucet to prevent the substrates from accumulating in the first place (sink overflow). Any IEM such as PKU, where treatment involves dietary restriction of the substrate (or precursors to the substrate) of the mutated enzyme, provides proof that the substrate is acting as a toxic metabolite.

In this review, based on this notion, we will present the case that there are numerous toxic metabolites that are not normally recognized as toxic metabolites but can clearly be demonstrated to act as such in the context of IEMs. Furthermore, these very metabolites appear to be involved in other nonhereditary contexts such as cancer and neurodegeneration, suggesting that there is value to studying these metabolites beyond the IEM. As studies on the same metabolite in these other diseases states often yield mechanistic insights into the biological effects of the accumulation of these metabolites, these studies may suggest new therapeutic angles for treating IEMs. Conversely, the clinical features of each IEM, such as the cell types which are affected, may also provide important hints as to why the metabolite can play a role in the other disorder. Thus, there is potentially high value in viewing IEMs from the perspective of the toxic metabolite that accumulates in each IEM and looking for what other disease contexts the toxic metabolite may be involved in. Importantly, the insights that are available from one disease context involving a toxic metabolite may be applied to understanding and treating the other. This review aims to illustrate this notion through two in-depth examples, MMA and hyperammonemia, and then briefly describe several other IEM/toxic metabolite contexts that similarly may share pathologies with other nonhereditary disorders. We will focus on amino acid metabolism disorders as examples, although the same concepts can likely be applied across the spectrum of metabolic disorders of other metabolic pathways, such as nucleic acid or lipid metabolism.

## 2. Methylmalonic Acidemia: An IEM and Toxic Metabolite with Broad Implications in Disease

### 2.1. Methylmalonic Acidemia

Methylmalonic acidemia is an autosomal recessive metabolic disorder characterized by elevated levels of methylmalonic acid (MMA) in the blood [19,20]. There are two main forms of the disease, one which involves only the loss of methylmalonyl-CoA mutase (MUT) activity, leading to elevated MMA, and the second which, in addition to MUT impairment, additionally involves the loss of Methionine Synthase (MTR), leading to homocysteine accumulation as well.

Both MUT and MTR are enzymes that require cobalamin, a copper-containing cofactor. Cobalamin is uptaken and released from the lysosome and processed in a series of common metabolic steps before undergoing divergent enzyme-catalyzed steps to form either adenosylcobalamin, a cofactor for MUT, or methylcobalamin, a cofactor for MTR. Thus, based on the specific gene that is mutated, either the function of MUT, MTR, or both could be affected [21].

The ‘isolated’ form of methylmalonic acidemia only involves MUT—either mutation in MUT itself or in the enzymes MMAA or MMAB, which are required specifically in the formation of adenosylcobalamin. Mutations in CD320 (transcobalamin receptor), involved in cobalamin import into cells, can also result in isolated methylmalonic acidemia.

**Figure 2:**
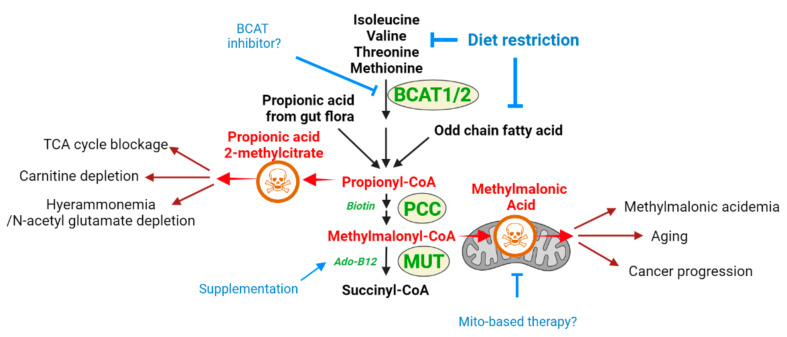
Schematic diagram of metabolic pathway containing toxic methylmalonyl-CoA and its toxicity mechanism and treatment options. In methylmalonic aciduria, branched-chain amino acids, fatty acids, or propionic acids produced by gut flora can feed into propionyl-CoA formation, leading to methylmalonyl-CoA production. The loss of MUT activity via mutations to the enzyme itself or via mutations to enzymes required to produce the adenosylcobalamin cofactor leads to an accumulation of methylmalonic acid. Methylmalonic acid appears to cause mitochondrial toxicity, and this mechanism may be at play in other disease contexts as well. Dietary restriction of branched-chain amino acids to restrict MMA production from above, or adenosylcobalamin supplementation to aid MUT activity, are current strategies. This diagram of MMA pathology suggests that a BCAT enzyme inhibitor, or therapies that aid mitochondrial function, may be considered as well. BCAT1/2: branched-chain amino acid aminotransferase1/2; PCC: propionyl-CoA carboxylase; MUT: methylmalonyl-CoA mutase.

The second form, combined methylmalonic acidemia with homocystinuria, is caused by mutations in steps common to both cobalamin forms, including MMACHC, MMADHC, and LMBRD1. This form can be diagnosed with the observation that both methylmalonic acid and homocysteine levels are elevated. Propionyl-CoA is formed as a breakdown intermediate in the catabolism of the branched-chain amino acids isoleucine and valine. It can also be formed as one of the fates of methionine or threonine catabolism, during the beta-oxidation of odd chain fatty acids, and even as a product of gut flora (Figure 2). Thus, using the kitchen sink analogy, isoleucine, valine, and additional factors can be considered upstream inputs that are ‘faucets’ forming propionyl-CoA and methylmalonyl-CoA, with MUT as the drain required to prevent the buildup of these metabolites. Furthermore, CoA is hydrolyzed from propionyl-CoA, and the propionyl group conjugates with carnitine, resulting in propionylcarnitine accumulation that is observed in conditions of MUT loss [22]. Both MMA and propionyl-CoA appear to be metabolites that are toxic when in excess, and both may mediate the toxicity seen in methylmalonic aciduria [22]. Indeed, propionyl-CoA accumulation is frequently observed in MMA, and priopionyl-CoA aciduria from PCC mutations is also a known IEM with severe pathologies [23].

The pathology of methylmalonic acidemia can vary greatly based on either the specific gene mutated or on the severity of the mutation, such as whether MUT mutations result in diminished or total loss of activity. For example, the total loss of MUT activity (mut^0^) can result in severe neonatal illness and mortality, while more benign forms of the disease with partial deficiency (mut^-^) exist [24]. Common manifestations of the disease include failure the thrive, hypotonia, developmental delay, and hematology abnormalities [21]. The main affected organ is the brain, with cognitive impairment and neural symptoms such as seizures, but it can affect a variety of organs outside of the CNS, such as the liver, kidneys, and pancreas [25]. As described in Section 2.1.2, therapeutic strategies involve preventing the accumulation of MMA and propionyl-CoA, demonstrating, like phenylketonuria, a ‘kitchen sink’ mechanic where reducing toxic metabolite accumulation is therapeutic.

#### 2.1.1. Methylmalonic Acid as a Toxic Metabolite Relevant to Multiple Disease States

It bears mentioning that methylmalonid acidemia may involve the toxic actions of additional toxic metabolites that accumulate in addition to MMA, including propionic acid and 2-methylcitrate, which are byproducts formed from propionyl-CoA, which sits further upstream of methylmalonyl-CoA and also accumulates in MMA-D (Figure 2) [26]. Furthermore, in the type of methylmalonic acidemia, which also involves homocysteine accumulation, homocysteine itself has toxic properties that may contribute to symptoms [27,28,29]. In this section, we will focus on MMA as an example of how insights into toxic metabolite mechanisms can inform future therapy strategies across different disease contexts involving MMA accumulation.

Methylmalonic acid is emerging as a toxic metabolite with important biological consequences outside of the methylmalonic academia. Methylmalonic acid is elevated during aging [30,31], which, at least in some cases, is caused by decreased vitamin B12 absorption and metabolism with aging [32,33,34]. The elevation of MMA in elderly people with deficiencies in B12 is associated with and may play causative roles in neuropsychiatric symptoms and macrocytic anemia [35]. Some studies have shed light on the toxic mechanisms for MMA, but our understanding of the detailed mechanisms is currently limited. It has been established that MMA is neurotoxic in culture models [36,37]. It is also well reported that mitochondrial function is negatively impacted, with mitochondrial uptake of MMA, inhibition of the TCA cycle, and electron transport being observed [37,38,39]. Inhibition of the succinate dehydrogenase complex activity [38,39,40] has been observed, and it is hypothesized that this inhibition may lead to energy production deficits in neurons. Further supporting the neurotoxic function of MMA, its elevation in B12-deficient patients with diabetes has been suggested to contribute to their neurodegeneration [41], and elevated MMA may be associated with neuropsychiatric disorders in elderly populations due to B12 deficiency [42]. As increases in methylmalonic acid appear to be a general feature of aging, even independently of B12 deficiencies [30,31], MMA may be viewed as a detrimental molecule exerting undesirable, neurotoxic effects as we age.

Aside from its CNS effects, MMA has recently been implicated in cancer contexts as well. While epidemiological risk analyses are not available, case reports of liver cancer in methylmalonic acidemia patients exist, raising the possibility that elevated MMA levels may be associated with increased cancer risk [43,44,45]. Further studies are required to establish MMA levels and cancer risk. Recently, MMA was shown to be an age-accumulating metabolic factor that increases the progression and aggressiveness of tumors [31]. MMA was found to induce SOX4, resulting in transcriptional reprogramming of cancer cells to endow them with malignant properties such as invasiveness and metastatic potential. Aside from providing a molecular explanation for how increased aging can increase cancer incidence and mortality, these findings provide important insights that may be relevant to understanding the downstream signaling effects that high levels of MMA might have in cells.

#### 2.1.2. Methylmalonic Acidemia and MMA as a Toxic Metabolite: What Does One Teach about the Other?

Current strategies for methylmalonic acidemia involve decreasing the formation of propionyl-CoA and methylmalonyl-CoA by reducing the various upstream inputs into their formation. One of the main strategies for treatment includes a specially managed diet that is restricted in valine/isoleucine/methionine/threonine, supporting that, indeed, the pathology of methylmalonic acidemia is due to a toxic accumulation of MMA [21,25]. However, such a diet is difficult to implement, and efficacy is variable [46,47]. Carnitine is also used as a therapy—the mechanism is not established, but benefits may be due to the formation of priopionylcarnitine and excretion by carnitine or due to a carnitine deficiency that can occur as a secondary downstream effect of reduced methylmalonyl-CoA mutase activity. Antibiotics may also be prescribed to reduce propionyl-CoA formation from gut flora [24]. Another therapeutic strategy for this disorder is adenosylcobalamin supplementation; while this is efficacious in cases with B12 deficiency, it is not effective in the majority of cases that involve defects in the MUT enzyme itself.

Viewing the methylmalonic acid disorder from the perspective of methylmalonic acid as a toxic metabolite suggests alternative strategies for treatment. The pathology can be viewed in a ‘kitchen sink’ analogy: the MUT enzyme, which metabolizes methylmalonyl-CoA, is the drain of the sink, and clogging the drain results in an ‘overflow’ of methylmalonyl-CoA, resulting in MMA accumulation. In this analogy, if toxicity occurs due to the accumulation of the metabolite, then inhibition of upstream enzymes, which lead to methylmalonyl-CoA production, is akin to turning off the faucet and should prevent MMA accumulation and pathology. Therefore, a possible therapeutic approach would be to target upstream enzymes of the branched amino acid degradation pathway to prevent the formation of MMA. While blocking the enzyme directly upstream of MMA production, propionyl-CoA carboxylase, would prevent MMA accumulation, it would result in propionyl-CoA accumulation (Figure 2). However, propionyl-CoA itself is also toxic and is a pathological agent in propionic acidemia [48]. Therefore, the ideal strategy may be to inhibit the pathway at a higher upstream step. One such candidate is the branched-chain aminotransferase (BCAT1/2) step, which is the initial step of the pathway and initiates the degradation of isoleucine/leucine/valine. Multiple BCAT inhibitors have been developed, including those with in vivo efficacy [49,50,51]. However, these were investigated for non-methylmalonic acidemia therapeutic contexts, such as diet-induced obesity or inflammatory disease, and to date, have not been explored in MMA-D. Such strategies may provide a means for reducing some of the sources of the production of propionyl-CoA/methylmalonyl-CoA/and MMA. The efficacy of such an approach remains to be examined, as BCAT inhibition would remove some sources of propionyl-CoA/MMA production, but not other inputs, such as odd-chain fatty acid beta-oxidation and propionyl-CoA from gut flora.

The studies on the MMA mechanism of toxicity that were carried out in the context of CNS and cancer may also provide insights into new therapeutic strategies for methylmalonic acidemia. For example, mitochondrial pathology is established as a downstream pathologic consequence of MMA; therapeutic strategies that target mitochondria, such as those that induce mitochondrial biogenesis or function [52], may be explored in this regard. Example strategies could include the administration of antioxidants such as coenzyme q10 or Vitamin E to decrease mitochondrial damage, as previously suggested for MMA and other metabolic disorders [53,54].

Any such strategies that decrease MMA production, or suppress the pathological events downstream of MMA accumulation, are potential therapies not only in methylmalonic acidemia but in the contexts of aging, neurodegeneration, and cancer, as MMA accumulation is involved in all of these contexts. For example, the BCAT inhibitors mentioned above may also have beneficial effects in decreasing the neurotoxic effects of elevated MMA in elderly diabetic patients. As already explored in the methylmalonic acidemia context, adenosylcobalamin supplementation may similarly aid in reducing MMA accumulation in the elderly, and indeed this facet has been explored in some epidemiological studies, although the overall findings are unclear [55]. The Gomes study [52] suggests that strategies to decrease MMA accumulation may be a means to decrease the risk of malignant cancer formation. These are just examples, and the central message is that as these diverse pathological states involve the same toxic metabolite, insights and therapeutic advancements gained in one context may also be highly beneficial in the other contexts. There is crosstalk value in considering mechanistic and therapeutic insights obtained in one MMA toxicity context with another, an approach that has not been carried out to this point.

### 2.2. Hyperammonemia and Ammonia Toxicity: A Second Example

**Figure 3:**
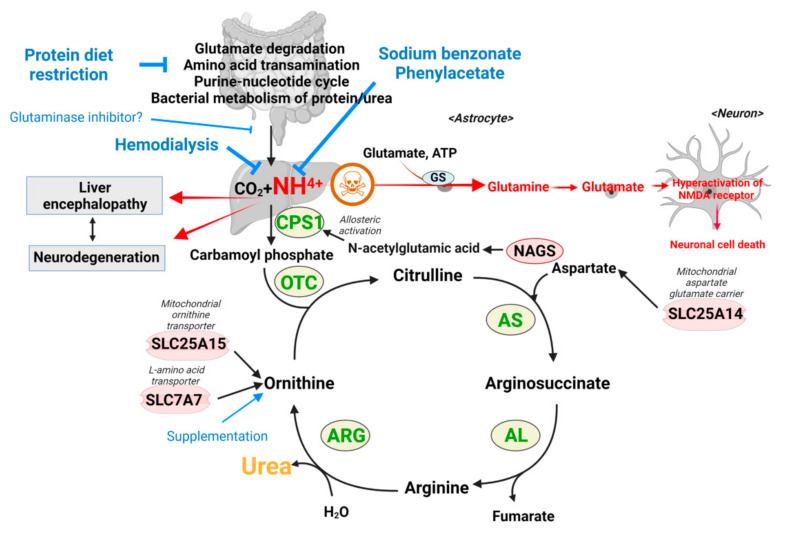
Schematic diagram of urea cycle that detoxifies ammonia, toxicity mechanism of ammonia, and therapeutic options for hyperammonemia. Ammonia is produced throughout the cells of the body in numerous metabolic processes such as transamination reactions, glutamate degradation, and nucleotide metabolism. These systemic levels of ammonia are cleared by the urea cycle in the liver so that the nitrogen is ultimately excreted in the form of urea. Impairing mutations in the urea cycle enzymes leads to an inability to clear ammonia, leading to the disorder of hyperammonemia. Dysfunction of the liver, such as due to cirrhosis, can similarly lead to secondary hyperammonemia. In these contexts, the prevailing model is that ammonia is used to produce high levels of glutamine in astrocytes, causing their swelling, and also leading to glutamate formation in neurons to trigger excitotoxicity. Current strategies involve protein restriction to decrease ammonia formation, sodium benzonate and phenylacetate treatment to ‘chelate’ ammonia, or supplementation to activate the urea cycle. GS: glutamine synthase; CPS1: carbamoyl phosphate synthetase; OTC: ornithine transcarbamylase; AS: argininosuccinate synthetase; AL: argininosuccinic acid lyase AL; ARG: arginase; NAGS: N-acetylglutamate synthase; SLC25A15: mitochondrial ornithine transporter 1; SLC7A7: y + L amino acid transporter 1; SLC25A14: mitochondrial aspartate glutamate carrier 2.

Hyperammonemia is a metabolic condition occurring either via inborn mutations and/or metabolic disturbance that is characterized by raised levels of ammonia (NH_3_) in the blood [56,57]. Patients have higher levels of ammonia (over 100–150 µM/L in neonates) compared with healthy individuals (45 ± 9 µM/L in healthy term infants, 50 µM/L in children, and less than 30 µM/L in adults) [58]. As excessive ammonia acts as a neurotoxin, the central nervous system is mainly affected, with symptoms including headache, vomiting, irritability, seizures, encephalopathy, coma, and even death, which are closely associated with neurological dysfunctions [58]. Normally, ammonia is produced in the small intestine and colon from multiple sources, including glutamine catabolism by glutaminase, amino acid transamination, purine–nucleotide cycle, and bacterial metabolism of protein/urea. This ammonia is transported to the liver, where the urea cycle is most active (Figure 3). Through the urea cycle, ammonia is processed to urea, a water-soluble metabolite, by a series of metabolic enzymes in the liver and then excreted via the kidneys.

Hyperammonemia can be classified into two different types: (i) that caused by a mutation of enzymes in the urea cycle is classified as primary hyperammonemia, and (ii) that caused by hepatic dysfunction or the inborn errors of intermediary metabolism, such as propionic acidemia, which indirectly leads to a dysfunctional urea cycle, is classified as secondary hyperammonemia [59].

(i) Primary hyperammonemia is caused by the defect of genes involved in the urea cycle. The first and rate-limiting step of the urea cycle is mediated by the carbamoyl phosphate synthetase (CPS), which converts ammonia to carbamoyl phosphate. Ornithine transcarbamylase (OTC) then converts carbamoyl phosphate to citrulline by donating the carbamoyl phosphate group to ornithine. Citrulline is further processed to arginosuccinate by argininosuccinate synthetase (AS) and then to arginine by argininosuccinic acid lyase (AL). As the last step, arginase (ARG) cleaves arginine to produce urea and ornithine, which are excreted or used for another cycle, respectively (Figure 3). Thus, the inactivation of any genes encoding enzymes for the urea cycle leads to perturbation of the urea cycle and accumulation of ammonia. In addition to the five enzymes in the urea cycle, defects of enzymes or transporters supporting the urea cycle can also cause hyperammonemia. For example, a defect of N-acetylglutamate synthase (NAGS), which produces N-acetylglutamic acid (NAcGlu), induces hyperammonemia as CPS activity is allosterically regulated by NAcGlu, a product of NAGS [60]. So, defects in NAGS lead to depletion of NAcGlu, leading to low CPS activity. As another example, hyperammonemia can be induced by defects in relevant transporters, such as mitochondrial ornithine transporter 1 (SLC25A15) and y + L amino acid transporter 1 (SLC7A7) [61,62]. As ornithine is a substrate for OTC enzyme in the urea cycle, the limited availability of ornithine because of defects of ornithine transport can lead to inactivation of the urea cycle. Lastly, the defect of SLC25A14 encoding mitochondrial aspartate glutamate carrier 2 (AGC2), also known as citrin, induces hyperammonemia by limiting the availability of aspartate, the substrate for AS enzyme reaction in the mitochondria [63]. Overall, mutations in any genes that lead to a defective urea cycle can promote primary hyperammonemia.
(ii) Hyperammonemia can also occur as a downstream consequence of other defects, and these cases are categorized as secondary hyperammonemia. In the liver, periportal hepatocytes process a vast amount of ammonia by expressing urea cycle enzymes, and perivenous hepatocytes metabolize ammonia with a low capacity by expressing glutamine synthetase (GS) as a back-up system [64]. Thus, the liver is critical in removing ammonia from the body, and reduced liver function and hepatotoxicity can also lead to hyperammonemia. Secondary hyperammonemia is diagnosed in various pathophysiological contexts, such as acute or chronic liver failure by hepatitis B viral infection, exposure to hepatotoxins, cirrhosis, and urinary tract infection by urease-producing bacteria [56,57]. Another form of secondary hyperammonemia can be observed in patients with propionic acidemia, methylmalonic acidemia, or glutaric acidemia type I. Propionic acidemia is a disease caused by propionyl-CoA carboxylase (PCC) deficiency [65,66]. PCC is an enzyme that processes propionyl-CoA; thus, the mutation in the PCCA or PCCB genes leads to the accumulation of propionyl-CoA. Since propionyl-CoA can inhibit NAGS [67], the urea cycle is slowed, which leads to hyperammonemia. The methylmalonyl-CoA accumulated in the patient with methylmalonic acidemia (see Section 2) also has the same inhibitory activity on NAGS as propionyl-CoA [67], so patients with methylmalonic acidemia showed hyperammonemia as well [67]. Hyperammonemia can be seen in cases and animal models of type I glutaric acidemia [68,69,70]. Thus, a variety of defects or insults can ultimately result in a pathological state of excess ammonia.

#### 2.2.1. Proposed Mechanisms of Ammonia as a Toxic Metabolite

Several toxicity mechanisms of ammonia have been suggested either in the context of models for primary or secondary hyperammonemia [71]. The most established model of the ammonia toxicity mechanism in the brain is neuronal cell death by the perturbed level of the neurotransmitter ‘glutamate’ (Figure 3). In the brain, astrocytes express glutamine synthase (GS), which catalyzes glutamine production using ammonia, glutamate, and ATP as substrates, and thus high systemic ammonia levels lead to high glutamine production in astrocytes (Figure 3) [72]. The excessive formation of glutamine by GS generates osmotic stress on astrocytes, which compromises astrocyte morphology and function and leads to astrocyte swelling [72,73]. The swelling of astrocytes by excessive ammonia induces the accumulation of glutamine extracellularly, which in turn are imported and converted to glutamate in neurons and secreted in synapses to trigger the activation of the N-methyl-D-aspartate (NMDA) receptor, and a cascade of neuronal death that is referred to as excitotoxicity [74,75,76]. Other receptors such as AMPA, mGluR, GABA, and benzodiazepine receptors are also affected by excessive glutamate [71].

Besides the excitotoxicity model, there are numerous findings that excessive ammonia can impact neuronal cells, with numerous mechanisms for toxicity proposed as follows.

(1) Excessive ammonia perturbs the serotonergic and cholinergic systems. The mouse-bearing mutation in the OTC gene, a hereditary hyperammonemia model, showed significant loss of cholinergic neurons, and the cholinergic neurons were damaged by ammonia in vitro culture [77,78], suggesting a direct toxic effect of ammonia on cholinergic neurons. In terms of underlying mechanisms that may cause toxicity, OTC-mutated mice showed elevated levels of tryptophan, a precursor for serotonin and serotonin metabolite [79], and altered expression of brain serotonin receptors, implying a disturbed serotoninergic system by hyperammonemia [80].
(2) A high level of ammonia disrupts ATP synthesis. The ammonia formed from glutamate deamination can perturb the H^+^ gradient across the mitochondrial inner membrane due to its alkaline property that binds to H^+^ [81]. Additionally, the expression and activity of cytochrome C oxidase, the electron transport chain enzyme, are significantly decreased in the hyperammonemia mouse model [82]. Therefore, the H^+^ gradient required for ATP synthesis is eliminated and thus leads to ATP depletion. Supporting this model, ATP concentration is lower in the brain of the OTC-mutated mouse [83].
(3) The TCA cycle is disrupted by excessive ammonia. The alpha-ketoglutarate dehydrogenase complex (KGDHC), which is required for producing alpha-ketoglutarate and NAD^+^, is inhibited by the pathological concentration of ammonia [84]. Thus, hyperammonemia induces defects in the TCA cycle.
(4) A high level of ammonia impairs ion homeostasis of astrocytes and neurons. The high level of ammonia perturbs astrocyte potassium buffering by increasing the concentration of extracellular potassium and hyperactivating the Na^+^-K^+^-2Cl^−^ cotransporter isoform 1 (NKCC1) in neurons [85]. So, genetic or pharmacological inhibition of NKCC1 has been shown to decrease the clinical and electrophysiological features of ammonia-induced neurotoxicity.
(5) Excessive ammonia impairs cerebral blood flow (CBF), which causes CBF autoregulation failure. Many studies have shown that patients with acute or chronic hyperammonemia suffer from brain edemas due to impaired CBF [86,87,88]. As hyperammonemia interferes with the metabolism of neurotransmitters and ion homeostasis and reduces the metabolic rate, as described above, ammonia accumulation leads to impaired CBF, which is closely regulated by cerebral metabolism.

However, the exact sequence and hierarchy of neuronal cell responses to high ammonia described are still unclear. Further studies will help determine the exact sequence/hierarchy of cellular events that occur as consequences of high ammonia in neurons.

#### 2.2.2. Ammonia as a Toxic Metabolite Relevant to Other Disease States

In agreement with its known neurotoxic properties, studies suggest that ammonia may play a contributory role in neurodegenerative disorders. For example, it was shown that ammonia levels in blood were significantly higher in patients with AD and ALS [89,90,91,92]. In addition, a high level of ammonia exposure has been shown to impair conditioned learning and memory [81,93] and inhibit motor function [94] in rats. The reason for increased ammonia levels may occur due to loss of blood–brain barrier integrity [91] and liver dysfunction, which may worsen with age, or even changes to gut microbiota [92]. Furthermore, the expression of glutamine synthetase is altered in a variety of age-dependent neurodegenerative contexts, such as AD and ALS [95]. In this manner, given the established toxicity of ammonia in the CNS, it can be hypothesized that ammonia can play a contributing role in neurodegeneration. Thus, more studies are necessary to figure out the clear linkage between neurodegenerative disease and ammonia toxicity.

Emerging evidence from various contexts has shown that increased ammonia also directly affects non-CNS cells, which raises the possibility that ammonia may play a contributory role in non-CNS diseases as well. For example, as kidney cells can take up ammonia from the circulation [58,96], in patients with liver disease, increased systemic ammonia results in damage to glomerular cells. In rats administered toxic levels of ammonia, activation of MAPK/ERKS was observed and postulated to mediate renal injury [97], which is a similar molecular response observed in astrocytes [98]. Another example of the direct effect of ammonia on non-CNS cells was observed in the hepatic stellate cell culture model. High ammonia levels induced the activation of NFkB and iNOS and upregulation of Toll-like receptor genes in the hepatic stellate cells [99]. Other contexts involving ammonia—accidental exposure or lung infection with *Coccidioides posadasii*, an ammonia-producing pathogen—demonstrate that the lung/respiratory tract is also susceptible to ammonia toxicity [100,101,102,103].

Collectively, these studies demonstrate that ammonia accumulation is relevant to multiple disease contexts and may exert pathogenic effects on multiple tissues in addition to the brain. The underlying mechanism of CNS toxicity appears to be well delineated in terms of glutamine overproduction and glutamate excitotoxicity, while the toxicity in non-brain cells is less complete but involves the aberrant activation of multiple signaling pathways.

#### 2.2.3. What Does One Inform about the Other?

The current treatment approaches vary depending on the degree of neurologic dysfunction and the level of acuity of liver failure. For acute hyperammonemia, the strategies are mainly aimed at restricting ammonia production or removing ammonia by (1) restricting proteins from the diet to decrease their catabolism that feeds into ammonia production (glucose with appropriate electrolytes is supplemented as an energy source); (2) injecting sodium benzoate and phenylacetate, which converts nitrogenous waste into benzoate-glycine and phenylacetate-glutamine rather than contributing to ammonia formation [104]; (3) the administration of N-carbamoyl-L-glutamic acid (NCG; a brand name Carbaglu), a synthetic analog of N-acetyl glutamate, that activates urea cycle by working as a cofactor for CPS1 [105]; and/or (4) hemodialysis to reduce the level of the ammonia in the blood. For the long-term management of urea cycle deficiency, the therapeutic strategies are aimed at diminishing chronic complications and sustaining normal development and growth by the combination of (1) a low-protein diet; (2) the supplementation of essential amino acids, vitamins, and minerals; (3) medication to elevate waste nitrogen excretion; and (4) the supplementation of arginine and/or citrulline [106].

As we have discussed how ammonia has neurotoxic properties, and its levels are elevated in contexts outside of primary and secondary hyperammonemia, such strategies used in hyperammonemia may be beneficial to these other contexts, such as AD and ALS. As ammonia-removal compounds have been shown to be safe and well-tolerated among patients with urea cycle defects, acute/chronic liver failure, and liver cancer [107], regimens based on benzoate and phenylacetate could be easily translated to use in other disease contexts which, as discussed, may at least partly involve ammonia elevation. Interestingly, a few studies have shown that sodium benzoate treatment protects dopaminergic neurons and astrocytes in animal models of Parkinson’s disease (PD) by stimulating the transcription of Glial-cell-line-derived neurotrophic factor (GDNF) in astrocytes [108,109]. Additionally, sodium benzoate improves memory and learning function in an animal model of AD by inhibiting neuronal cell apoptosis, glial activation, and Aβ burden [110,111]. These studies of the utilization of sodium benzoate in animal models of PD and AD support the notion that the treatment strategies of hyperammonemia could be translated to other disease models, though it is unclear yet if the beneficial effect of sodium benzoate on PD and AD is indeed mediated by its nitrogen-scavenging activity.

Turning focus back to hyperammonemia, the studies in Section 2.2.2, which examined the downstream mechanisms engaged by high levels of ammonia, suggest alternative therapeutic strategies to counteract the downstream effects of ammonia rather than prevent ammonia production. First of all, an NMDA receptor antagonist or GS inhibitor could be considered a therapeutic drug for hyperammonemia because the most established model of the working mechanism of ammonia toxicity is the activation of the NMDA receptor, which is initiated by the GS enzyme in astrocyte (Figure 3). Currently, several clinically approved NMDA receptors that have been developed for other psychiatry diseases and GS inhibitors are available [73,112]. Indeed, L-methionine-S,R-sulfoximine, a GS inhibitor, has been shown to attenuate the neuropathological changes in acutely hyperammonemic rats [73]. As another approach for treating hyperammonemia, bumetanide, an inhibitor of the Na^+^-K^+^-2Cl^−^ cotransporter, could be used for alleviating the clinical and electrophysiological features of neurotoxicity due to excessive ammonia, which hyperactivates the Na^+^-K^+^-2Cl^−^ cotransporter [85]. Additionally, restoring the TCA cycle and ATP production could be considered a strategy to treat hyperammonemia-induced neuronal cell death [84]. As an example of the fueling strategy, acetyl-L-carnitine has been shown to recover the cerebral energy deficits caused by excessive ammonia. Carnitine promotes the TCA cycle, which leads to ATP production through shuttling acyl-CoA across the mitochondrial membrane, where acyl-CoA undergoes beta-oxidation. Thus, while there are well-established and effective clinical strategies to manage hyperammonemia, these studies suggest supplemental strategies to minimize the toxicity of ammonia. Furthermore, such strategies directly addressing neurotoxic mechanisms may be considered in the contexts of AD and ALS as well.

We can also learn how to manage the inborn error urea cycle from the therapeutic strategies for secondary hyperammonemia [85]. Various methods to lower systemic ammonia levels have been explored for secondary hyperammonemia. First, to reduce the absorption of ammonia and bacterial production of ammonia in the intestine, non-absorbable disaccharides such as lactulose [85] or antibiotics such as Rifaximin [85] and Neomycin [85] were used to limit intestinal bacteria growth. Second, to improve the ammonia-removal capacity, ammonia-utilizing metabolic pathways such as glutamine synthesis and the urea cycle were boosted by adding the salts L-ornithine L-aspartate or L-ornithine phenylacetate, which fuel the urea cycle of residual hepatocytes [85] and activates glutamine synthetase activity of the muscle [85]. Third, inhibitors of glutaminase, which generates ammonia, have also been considered a treatment option for hyperammonemia since they can restrict ammonia-forming glutaminolysis [113,114]. Indeed, metformin and thiourea derivative THDP-17, which partially inhibits glutaminases, reduced glutamine production [85]. Fourth, bioartificial liver support systems have been developed for patients suffering from acute and chronic liver failure. The bioartificial liver support system is based on conventional hemodialysis, which is combined with filtration via hollow-fiber cartridges containing hepatoblastoma cells with the capacity to detoxify ammonia [85]. There are obvious limitations to such strategies—for example, strategies for activating the urea cycle may not be effective in primary hyperammonemia caused by urea cycle enzyme defects. Nonetheless, these studies suggest that there are multiple therapeutic strategies to decrease ammonia that should be considered not only for IEM hyperammonemia but to minimize ammonia neurotoxicity that may contribute to the pathology of neurodegenerative disorders such as AD.

## 3. Toxic Metabolites Revealed in Inborn Metabolic Disorders May Have Broad Roles in Pathology

The toxic metabolite accumulation model described in methylmalonic acidemia and hyperammonemia appears hold true for the majority of amino-acid-breakdown disorders. Evidence of toxicity of the accumulated substrates, diet-restriction-based therapeutic strategies designed to decrease metabolite production, and possible involvement of the metabolite in other disorders is evident in the literature (Table 1), as briefly described here. These examples are covered in excellent reviews elsewhere [85] and are only briefly discussed here to exemplify how multiple amino acid disorders clearly involve the toxic accumulation of certain metabolites, raising an open question of whether such metabolites could contribute to other pathological contexts.

Tyrosinemia. Type II of the disorder involves impairment in the first step of tyrosine degradation, tyrosine aminotransferase (TAT), causing tyrosine accumulation. Type III is caused by impairment in the 4-hydroxyphenylpyruvate dioxygenase (HPD) enzyme, which is one step downstream [115]. Type I tyrosinemia involves the loss of fumarylacetoacetate hydrolase (FAH), which converts fumarylacetoacetate to fumarate and acetoacetate a few steps further downstream of the tyrosine degradation pathway. While these subtypes vary in disease symptoms and prognoses, in all subtypes, dietary restriction of tyrosine is prescribed, indicating that tyrosine or breakdown intermediates that accumulate are toxic. Notably, the administration of an HPD inhibitor, 2-(2-nitro-4-trifluoromethylbenzoyl)-1,3-cyclohexanedione (NTBC), has been shown to alleviate symptoms of type I tyrosinemia [116,117], suggesting that the accumulation of fumarylacetoacetate might have higher toxic potential than the accumulation of other upstream metabolites in the tyrosine degradation pathway. Adverse effects of excess tyrosine consumption per se have been noted, raising the possibility of tyrosine contributing to other pathologies [118,119].

Nonketotic hyperglycinemia (NKH). NKH is caused by loss of function mutations in enzymes such as glycine decarboxylase (GLDC), which comprise the glycine cleavage system, which breaks down glycine. A toxic buildup of glycine results in severe encephalopathy, intellectual disability, and typically progresses to death. While no successful treatment exists for severe forms of NKH, dietary restriction of glycine or glycine ‘chelation’ via sodium benzoate improves outcomes in milder cases [120,121,122], indicating a toxic glycine accumulation model for pathology. Since glycine is an allosteric activator of the NMDA receptor of neurons, glycine accumulation in the patient with hyperglycinemia overstimulates the NMDA receptor, which has been associated with seizures and developmental delays. Thus, inhibitors of the NMDA receptor, such as dextromethorphan or ketamine, have been shown to decrease seizure propensity and improve neurocognitive outcomes in combination with sodium benzoate [120].

While these are just a few examples, they raise the possibility that various other inborn errors of metabolism may involve the accumulation of toxic metabolites that may also play roles in other disease contexts and thus have important implications in understanding the pathology of these conditions. Other such examples of disorders involving toxic metabolite accumulation are listed in Table 1.

**Table 1:** List of IEMs caused by toxic metabolite accumulation and their toxicity mechanism and current treatment options.

Name of Disease	Associated Genes	Accumulated Toxic Metabolites	Toxicity Mechanism	Current Treatment Options
Methylmalonic acidemia	MUT	Methylmalonic, hydroxypropionic, methylcitric acids	Energy production deficits via inhibition of TCA cycle and electron transport [36,38,39,40,123]	Restrict branched-chain amino acids Carnitine supplementation Adenosylcobalamin supplementation
Primary Hyperammonemia	NAGS, CPS 1, OTC, ASS, SLC25A1, SLC25A14, SLC7A7	Ammonia	Activation of NMDA receptor by increased glutamate, which is from astrocytes [72] Generation of oxidative stress, inhibition of respiratory chain (energy defects) [74,75,76]	Restrict protein diet Injection of sodium benzoate and phenylacetate Hemodialysis [58] Supplementation of N-carbamoyl-L-glutamic acid (NCG) [105]
Phenylketonuria	PAH	Phenylalanine	Restricted transportation of tryptophan and tyrosine at BBB due to high prevalence of phenylalanine occupying L-amino acid transporters [124] Self-assembly of phenylalanine, which is linked to amyloid formation [125]	Restrict protein diet Supplementation of phenylalanine-free amino acid mixture Supplementation of a combination of tyrosine, L-dopa, and 5-hydroxytrytophan [126] Supplementation of tetrahydrobiopterin, which stimulates PAH activity [15]
Isovaleric acidemia	IVD	Isocaleryl-CoA derivatives: isovaleric acid, hydroxyisovaleric acid	Inhibition of Na(+), K(+)-ATPase activity via ROS production [127]	Leucin restriction, L-carnitine, and/or glycine supplementation [128,129]
Tyrosinemia type 1	FAH	Fumarylacetoacetate, succinylacetone	Induction of cytochrome c release [117]	Tyrosine restriction Administration of 2-(2-nitro-4-trifluoromethylbenzoyl)-1,3-cyclohexanedione (NTBC), the inhibitor for 4-hydroxyphenylpyruvate dioxygenase (HPD), upstream enzyme of fumarylactoacetate [116,130]
Maple syrup urine disease	BCKDHA, BCKDHB, DBT	Leucine, isoleucine, and valine	Dysregulated amino and organic acids in brain [131]	Dietary restriction of BCAAs Liver transplantation [131]
Glycine encephalopathy	GLDC	Glycine-> Methylglyoxal	Damaged macromolecules (nucleic acids, proteins) as a reactive metabolite [132]	Administration of sodium benzoate to reduce plasma concentration of glycine Blockade of overstimulated NMDA receptors [133]
Homocystinuria	CBS MTR	Homocysteine	Induction of oxidative stress, nitrosylation, homocysteinylation, and hypomethylation [134]	Methionine restriction Supplementation with vitamin B6, a cofactor for BHMT that can metabolize homocysteine [135]
Propionic academia	PCCA, PCCB	Propionyl-carnitine	Unknown	Restrict protein intake Remove toxic compounds with nitrogen-scavenger medications, extracorporeal detoxification, and/or intravenous carnitine Hemodialysis [48]
Cystinuria	SLC3A1, SLC7A9	Cystine	Formation of cystine stones [136]	Increase fluid intake to increase cystine solubility Administration of thiol drugs [136]
3-Hydroxy-3-methylglutaryl-coenzyme A lyase deficiency	HMGCL	3-hydroxy-3- methylglutaric acid, 3-methylglutaric acid, 3-hydroxyisovaleric acids	Induction of oxidative stress Disruption of bioenergetics, dynamics, ER-mitochondria communication, and signaling pathways [137] Induction of DNA damage [138]	Restrict leucine diet Supplementary glucose [139,140]
Hyperprolinemia	PRODH ALDH4A1	Proline	Excitotoxin [141] Reduction in glutamate uptake, Na(+), K(+)-ATPase activity, ATP levels Increased lipid peroxidation [142]	Restriction of dietary proline [143]
Hyperlysinemia	AASS	Lysine, Pipecolic acid	Unknown	Dietary restriction of lysine [144]

Full name of enzymes—MUT: Methylmalonyl-CoA Mutase; NAGS: N-Acetylglutamate Synthase; CPS 1: Carbamoyl-Phosphate Synthase 1; OTC: Ornithine Transcarbamylase; ASS: Argininosuccinate Synthase 1; PAH: Phenylalanine Hydroxylase; IVD: Isovaleryl-CoA Dehydrogenase; FAH: Fumarylacetoacetate Hydrolase; BCKDHA/BCKDHB: Branched-Chain Keto Acid Dehydrogenase E1 Subunit Alpha/Beta; DBT: Dihydrolipoamide Branched-Chain Transacylase E2; GLDC: Glycine Decarboxylase; CBS: Cystathionine Beta-Synthase; MTR: 5-Methyltetrahydrofolate-Homocysteine Methyltransferase; PCCA/PCCB: Propionyl-CoA Carboxylase Subunit Alpha/Beta; HMGCL: 3-Hydroxy-3-Methylglutaryl-CoA Lyase; PRODH: Proline Dehydrogenase 1; ALDH4A1: Aldehyde Dehydrogenase 4 Family Member A1; AASS: Aminoadipate-Semialdehyde Synthase.

Homocystinuria. Homocysteine is an intermediate in methionine metabolism and accumulates due to the loss of cystathionine beta-synthase (CBS) [135,145] or methionine synthase (MTR) [146,147], which metabolizes homocysteine to cystathionine or methionine, respectively. In contrast to most amino acid disorders in which the brain is the primary affected organ, homocystinuria involves multiple organs, including the eye, skeletal system, and the brain [135]. Treatment for homocystinuria shows partial efficacy and involves methionine restriction and supplementation with vitamin B6, which is a cofactor for another enzyme, Betaine-homocysteine S-methyltransferase (BHMT), which can metabolize homocysteine [135]. Thus, homocystinuria is also a toxic accumulation disorder.

As described in methylmalonic acidemia and hyperammonemia, toxic metabolic intermediates in these various disorders appear to play roles in other pathological states as well. Elevated homocysteine levels are a risk factor for vascular diseases [148,149,150], Alzheimer’s [151,152], as well as cancer [153,154]. Homocysteine’s role in some of these pathologies may involve the formation of reactive oxygen species or inhibition of transmethylation reactions [155,156]. Homocysteine can form fibrillar assemblies that can crosstalk with amyloid fibrils, providing a possible mechanism for involvement in Alzheimer’s [157]. Along similar lines, phenylalanine also forms amyloid-like fibrillar deposits [125], which not only may explain some of the neuropathological symptoms of PKU but also suggest that elevated levels of phenylalanine could contribute to AD and other neurodegenerative disorders. Along these lines, there are links between tyrosine metabolism and tyrosine hydroxylase and PD [158,159], as well as hyperlysinemia and hyperprolinemia with AD [160].

## 4. Conclusions

As we have discussed in this review, inborn errors of metabolism clearly demonstrate the pathological consequences that occur when certain metabolites accumulate to toxic levels. The current treatments for most of these disorders are either only partially effective, difficult to implement, or nonexistent. Here, we propose an approach to view the disorder from the perspective of the toxic metabolite to develop effective therapies, which may be more feasible than the replacement of the enzyme activity that is lost due to the underlying mutation(s). First, limiting the production of the toxic metabolite is an attractive strategy that is already implemented in multiple amino acid breakdown disorders through dietary restriction of the toxic metabolite or its upstream precursors (e.g., phenylketonuria, methylmalonic acidemia). Theoretically, the inhibition of the upstream enzymes that are required to produce the toxic metabolite, such as inhibition of BCAT1 in methylmalonic acidemia, would provide an alternative method that is more feasible than preventing the intake of specific amino acids from the diet. Metabolic enzymes are inherently druggable, and in some cases, such as BCAT1, inhibitor candidates are already available, having been developed for other purposes.

As also outlined here, these toxic metabolites appear to be involved in contexts outside of their inborn metabolic disorder, such as aging, cancer, and neurodegeneration. This overlap provides useful opportunities to utilize knowledge gained from one disease context for the understanding and treatment of another disease context. For example, dietary restriction methods that have been proven to be effective in preventing the accumulation and pathology of a metabolic disorder may be explored as a preventative or auxiliary method to slow the progression of a neurodegenerative disorder in which the toxic metabolite may have a contributing role. As another example, studies of a toxic metabolite such as ammonia in cancer cells often yield mechanistic insights into the cellular consequences of toxic metabolite excess, and this information can be used to design therapies to counteract such consequences in the context of metabolic disorders. We discussed multiple examples of such overlaps and the therapeutic opportunities that they raise in the examples of methylmalonic acidemia and hyperammonemia, and we believe that many other examples will be uncovered where various endogenously formed metabolites that are shown to be toxic at high levels in metabolic disorders will also be shown to contribute to pathologies in other contexts, such as cancer.

Finally, the role of such toxic metabolites, not only as causative agents in metabolic disorders but also aging, cancer, and neurologic disorders, illustrates how metabolism requires tight control, where an imbalance between the production and degradation of a metabolite can result in disastrous consequences for the patient. A picture is emerging where metabolic imbalance is not only a disorder of inborn errors of metabolism but may have a degree of contribution to a wide range of pathological states.
